# Supplementary material for: Intragenic epistasis in couch potato shapes life history traits and climatic adaptation in Drosophila melanogaster
Source: bioRxiv. 2026 Oct 11:2026.10.09.758124. Preprint. [Version 1] doi: 10.64898/2026.10.09.758124 (PMC13692285; doi:10.64898/2026.10.09.758124)
Supplement: Supplementary file 1 [file NIHPP2026.10.09.758124v1-supplement-2.pdf]

bioRxiv preprint doi: https://doi.org/10.64898/2026.10.09.758124; this version posted October 11, 2026. The copyright holder for this preprint (which was not certified by peer review) is the author/funder, who has granted bioRxiv a license to display the preprint in perpetuity. It is made available under a CC-BY 4.0 International license.

Table S1. *cpo* SNPs that are clinal with a q-value (CQ) of <10^{-4} and their allele frequency at sampled populations along a latitudinal cline. Focal alleles are highlighted in bold.

Position on 3R	CQ	Allele frequency at latitude (ºN)								
		25.47	25.47	30.32	33.39	35.77	40	40	44.03	44.03
17939248	6.90×10^{-05}	0.48	0.36	0.32	0.14	0.10	0.04	0.16	0.05	0.04
17940255	3.00×10^{-06}	0.19	0.09	0.23	0.26	0.47	0.37	0.35	0.59	0.58
17940301	3.40×10^{-05}	0.25	0.14	0.21	0.25	0.34	0.38	0.30	0.62	0.40
17940310	4.80×10^{-05}	0.44	0.53	0.49	0.43	0.39	0.10	0.13	0.07	0.27
17943485	3.00×10^{-06}	0.51	0.62	0.42	0.36	0.19	0.14	0.08	0.09	0.13
17947180	9.00×10^{-06}	0.67	0.68	0.73	0.51	0.42	0.39	0.39	0.23	0.22
17955967	7.90×10^{-05}	0.44	0.67	0.44	0.20	0.17	0.18	0.16	0.12	0.10
17955974	4.20×10^{-05}	0.37	0.52	0.43	0.14	0.06	0.08	0.12	0.04	0.03
17956579	1.90×10^{-05}	0.47	0.43	0.40	0.32	0.20	0.13	0.21	0.07	0.10
17956683	5.00×10^{-06}	0.47	0.46	0.55	0.28	0.11	0.14	0.16	0.04	0.10
17956684	1.00×10^{-06}	0.46	0.48	0.54	0.27	0.10	0.13	0.14	0.02	0.10
17956708	1.00×10^{-06}	0.49	0.53	0.54	0.32	0.15	0.14	0.29	0.06	0.13
17956748	3.10×10^{-05}	0.54	0.48	0.51	0.33	0.16	0.12	0.33	0.13	0.19
17956840	4.70×10^{-05}	0.51	0.67	0.47	0.28	0.24	0.13	0.21	0.10	0.41
17956850	4.90×10^{-05}	0.49	0.62	0.45	0.26	0.27	0.13	0.18	0.09	0.41
17957171	3.30×10^{-05}	0.60	0.65	0.76	0.53	0.27	0.32	0.43	0.30	0.19
17957175	3.60×10^{-05}	0.60	0.67	0.76	0.50	0.26	0.30	0.41	0.31	0.21

bioRxiv preprint doi: https://doi.org/10.64898/2026.10.09.758124; this version posted October 11, 2026. The copyright holder for this preprint (which was not certified by peer review) is the author/funder, who has granted bioRxiv a license to display the preprint in perpetuity. It is made available under a CC-BY 4.0 International license.

17957180	4.90 x10^{-05}	0.62	0.67	0.74	0.50	0.32	0.30	0.41	0.31	0.19
17957310	6.00 x10^{-05}	0.59	0.53	0.45	0.40	0.39	0.12	0.13	0.15	0.13
17958784	4.70 x10^{-05}	0.44	0.47	0.34	0.11	0.10	0.06	0.13	0.05	0.17
17958792	5.50 x10^{-05}	0.46	0.52	0.39	0.13	0.13	0.09	0.22	0.07	0.23
17959043	6.00 x10^{-06}	0.47	0.56	0.38	0.14	0.13	0.03	0.16	0.05	0.07
17963438	2.00 x10^{-05}	0.76	0.91	0.84	0.66	0.70	0.84	0.73	0.43	0.50
17963448	2.00 x10^{-06}	0.82	0.93	0.86	0.68	0.67	0.84	0.73	0.43	0.47
17963734	7.40 x10^{-05}	0.43	0.38	0.34	0.19	0.18	0.08	0.12	0.06	0.04
17964408	**<1** x10^{-07}	**0.82**	**0.75**	**0.57**	**0.59**	**0.51**	**0.54**	**0.35**	**0.13**	**0.56**
17965538	6.10 x10^{-05}	0.41	0.26	0.22	0.37	0.56	0.55	0.46	0.68	0.61
17965558	**<1** x10^{-07}	**0.20**	**0.32**	**0.43**	**0.47**	**0.63**	**0.71**	**0.67**	**0.81**	**0.80**
17965930	2.50 x10^{-05}	0.31	0.43	0.44	0.72	0.59	0.70	0.73	0.79	0.71
17965958	1.40 x10^{-05}	0.21	0.49	0.50	0.80	0.63	0.70	0.70	0.83	0.75
17967866	**2.00** x10^{-06}	**0.23**	**0.03**	**0.12**	**0.27**	**0.50**	**0.41**	**0.42**	**0.61**	**0.52**
17970970	1.10 x10^{-05}	0.65	0.71	0.70	0.66	0.54	0.35	0.56	0.25	0.35
17984073	3.30 x10^{-05}	0.13	0.21	0.40	0.47	0.45	0.45	0.40	0.68	0.75
17984172	7.00 x10^{-06}	0.22	0.10	0.35	0.68	0.50	0.36	0.43	0.73	0.68
17986523	9.10 x10^{-05}	0.01	0.08	0.14	0.17	0.37	0.40	0.26	0.36	0.47
17989198	5.30 x10^{-05}	0.62	0.56	0.58	0.41	0.24	0.30	0.33	0.24	0.19
17994067	5.70 x10^{-05}	0.08	0.05	0.19	0.25	0.30	0.23	0.35	0.44	0.44

bioRxiv preprint doi: https://doi.org/10.64898/2026.10.09.758124; this version posted October 11, 2026. The copyright holder for this preprint (which was not certified by peer review) is the author/funder, who has granted bioRxiv a license to display the preprint in perpetuity. It is made available under a CC-BY 4.0 International license.

17997305	4.00×10^{-06}	0.85	0.76	0.61	0.46	0.35	0.59	0.47	0.29	0.29
17998827	8.00×10^{-06}	0.61	0.50	0.42	0.37	0.12	0.26	0.21	0.07	0.24
18000687	4.60×10^{-05}	0.72	0.91	0.75	0.61	0.42	0.52	0.60	0.28	0.71
18001545	7.30×10^{-05}	0.43	0.47	0.43	0.25	0.21	0.13	0.08	0.05	0.11
18013015	7.00×10^{-06}	0.67	0.62	0.62	0.59	0.18	0.50	0.62	0.14	0.21
18013084	$<1 \times 10^{-07}$	0.59	0.58	0.41	0.39	0.16	0.31	0.35	0.07	0.08
18013126	$<1 \times 10^{-07}$	0.59	0.64	0.45	0.36	0.09	0.34	0.34	0.11	0.10
18019905	2.60×10^{-05}	0.89	0.75	0.72	0.50	0.57	0.28	0.47	0.36	0.19

bioRxiv preprint doi: https://doi.org/10.64898/2026.10.09.758124; this version posted October 11, 2026. The copyright holder for this preprint (which was not certified by peer review) is the author/funder, who has granted bioRxiv a license to display the preprint in perpetuity. It is made available under a CC-BY 4.0 International license.

Table S2. Drosophila Genetics Reference Panel lines (RAL) used to initiate each Recombinant Outbred Population. Bloomington Drosophila Stock Center number is indicated when available.

ROP	RAL	BDSC#
cpo^{TTA}	RAL-21	28122
	RAL-42	28127
	RAL-93	28137
	RAL-239	28161
	RAL-320	29654
	RAL-325	NA
	RAL-399	25192
cpo^{TTT}	RAL-75	28132
	RAL-91	28136
	RAL-181	28151
	RAL-235	28275
	RAL-356	28178
	RAL-359	28179
	RAL-426	28196
cpo^{TGT}	RAL-149	28145
	RAL-370	28182

bioRxiv preprint doi: https://doi.org/10.64898/2026.10.09.758124; this version posted October 11, 2026. The copyright holder for this preprint (which was not certified by peer review) is the author/funder, who has granted bioRxiv a license to display the preprint in perpetuity. It is made available under a CC-BY 4.0 International license.

	RAL-790	28232
	RAL-818	28241
	RAL-859	25210
	RAL-890	28257
	RAL-894	NA
cpo^{TGA}	RAL-57	29652
	RAL-83	28134
	RAL-217	28154
	RAL-367	NA
	RAL-371	28183
	RAL-492	28203
	RAL-837	28246
cpo^{CTA}	RAL-177	28150
	RAL-318	28168
	RAL-381	28188
	RAL-531	28207
	RAL-757	28226
	RAL-738	28223
	RAL-805	28237
cpo^{CTT}	RAL-40	29651

bioRxiv preprint doi: https://doi.org/10.64898/2026.10.09.758124; this version posted October 11, 2026. The copyright holder for this preprint (which was not certified by peer review) is the author/funder, who has granted bioRxiv a license to display the preprint in perpetuity. It is made available under a CC-BY 4.0 International license.

	RAL-109	28140
	RAL-195	28153
	RAL-227	28156
	RAL-228	28157
	RAL-229	29653
	RAL-233	NA
cpo^{CGT}	RAL-26	28123
	RAL-105	28139
	RAL-142	28144
	RAL-338	28173
	RAL-362	25187
	RAL-386	28192
	RAL-406	29657

bioRxiv preprint doi: https://doi.org/10.64898/2026.10.09.758124; this version posted October 11, 2026. The copyright holder for this preprint (which was not certified by peer review) is the author/funder, who has granted bioRxiv a license to display the preprint in perpetuity. It is made available under a CC-BY 4.0 International license.

Table S3. Effects of individual focal SNPs (*cpo*^{4408}, *cpo*^{5558}, *cpo*^{7866}) on each fitness-related trait, from the additive model used to generate the null prediction for each three-locus genotype combination (see Methods). For each locus, the significance test is reported alongside the marginal mean ± SE for the temperate and tropical allele at that locus, estimated from the same fitted model. * p<0.05, ** p<0.01, *** p<0.001.

Proportion dormant

	df	Chisq	Pr		temperate	tropical
cpo^{4408}	1	0.376	0.5395		(T): 0.71 ± 0.05	(C): 0.75 ± 0.05
cpo^{5558}	1	28.210	1.09e-7	***	(T): 0.87 ± 0.03	(G): 0.53 ± 0.05
cpo^{7866}	1	0.044	0.8342		(A): 0.73 ± 0.05	(T): 0.74 ± 0.04

Chill recovery time (min)

	df	F	Pr	temperate	tropical
cpo^{4408}	1	3.600	0.0619	(T): 33.21 ± 2.29	(C): 39.71 ± 2.53
cpo^{5558}	1	0.459	0.5002	(T): 35.32 ± 2.45	(G): 37.61 ± 2.35
cpo^{7866}	1	3.233	0.0765	(A): 39.66 ± 2.89	(T): 33.26 ± 1.95

Lipid mass content (ng)

	df	F	Pr	temperate	tropical
cpo^{4408}	1	1.507	0.2206	(T): 65.38 ± 2.55	(C): 60.32 ± 3.25
cpo^{5558}	1	3.863	0.0503	(T): 66.87 ± 2.60	(G): 58.83 ± 3.20
cpo^{7866}	1	0.412	0.5215	(A): 64.17 ± 3.26	(T): 61.53 ± 2.54

bioRxiv preprint doi: https://doi.org/10.64898/2026.10.09.758124; this version posted October 11, 2026. The copyright holder for this preprint (which was not certified by peer review) is the author/funder, who has granted bioRxiv a license to display the preprint in perpetuity. It is made available under a CC-BY 4.0 International license.

Starvation survival time (hours)

	df	F	Pr		temperate	tropical
cpo^{4408}	1	1.851	0.1784		(T): 83.71 ± 1.84	(C): 87.66 ± 2.25
cpo^{5558}	1	16.317	0.0001	***	(T): 91.57 ± 1.84	(G): 79.81 ± 2.26
cpo^{7866}	1	0.023	0.8797		(A): 85.47 ± 2.26	(T): 85.91 ± 1.84

Heat knockdown time (min)

	df	F	Pr		temperate	tropical
cpo^{4408}	1	1.749	0.1869		(T): 77.81 ± 2.08	(C): 73.55 ± 2.40
cpo^{5558}	1	0.109	0.7415		(T): 75.15 ± 2.05	(G): 76.21 ± 2.43
cpo^{7866}	1	1.592	0.2079		(A): 77.70 ± 2.39	(T): 73.66 ± 2.08

Lifespan (days)

	df	F	Pr		temperate	tropical
cpo^{4408}	1	25.716	3.54e-6	***	(T): 29.42 ± 0.51	(C): 33.52 ± 0.63
cpo^{5558}	1	6.536	0.0129	*	(T): 32.51 ± 0.51	(G): 30.43 ± 0.64
cpo^{7866}	1	27.180	2.09e-6	***	(A): 29.35 ± 0.64	(T): 33.59 ± 0.51

Development time (hours)

	df	F	Pr		temperate	tropical
cpo^{4408}	1	0.799	0.3746		(T): 222.49 ± 0.77	(C): 223.57 ± 0.94
cpo^{5558}	1	1.075	0.3035		(T): 223.66 ± 0.77	(G): 222.40 ± 0.94
cpo^{7866}	1	10.471	0.0019	**	(A): 221.07 ± 0.94	(T): 224.99 ± 0.77

bioRxiv preprint doi: https://doi.org/10.64898/2026.10.09.758124; this version posted October 11, 2026. The copyright holder for this preprint (which was not certified by peer review) is the author/funder, who has granted bioRxiv a license to display the preprint in perpetuity. It is made available under a CC-BY 4.0 International license.

Fecundity (number of eggs)

	df	F	Pr	temperate	tropical
cpo^{4408}	1	1.983	0.1645	(T): 32.36 ± 2.97	(C): 38.75 ± 3.55
cpo^{5558}	1	1.224	0.2733	(T): 32.97 ± 2.87	(G): 38.14 ± 3.71
cpo^{7866}	1	1.277	0.2632	(A): 32.86 ± 3.80	(T): 38.25 ± 2.83

Relative *cpo* expression ($2^{-\Delta Cq}$)

	df	F	Pr	temperate	tropical
cpo^{4408}	1	1.803	0.1981	(T): 0.0448 ± 0.0070	(C): 0.0604 ± 0.0092
cpo^{5558}	1	0.472	0.5018	(T): 0.0486 ± 0.0070	(G): 0.0566 ± 0.0092
cpo^{7866}	1	0.532	0.4765	(A): 0.0485 ± 0.0086	(T): 0.0566 ± 0.0074

bioRxiv preprint doi: https://doi.org/10.64898/2026.10.09.758124; this version posted October 11, 2026. The copyright holder for this preprint (which was not certified by peer review) is the author/funder, who has granted bioRxiv a license to display the preprint in perpetuity. It is made available under a CC-BY 4.0 International license.

Table S4. Additive null prediction and observed trait value for each cpo three-locus genotype combination in recombinant outbred populations (ROPs). Prediction is the mean of the three marginal single-locus emmeans ± propagated SE; Observation is the ROP emmean ± SE. Deviation is observed minus predicted. The *cpo*^{CGA} combination is not shown, as it was too rare in the DGRP to establish a ROP.

Proportion dormant

cpo haplotype	Prediction ± SE	Observation ± SE	Deviation
CGT	0.674 ± 0.028	0.605 ± 0.079	-0.069
CTA	0.784 ± 0.025	0.867 ± 0.051	0.082
CTT	0.789 ± 0.023	0.864 ± 0.052	0.075
TGA	0.655 ± 0.030	0.400 ± 0.089	-0.255
TGT	0.660 ± 0.028	0.537 ± 0.077	-0.124
TTT	0.775 ± 0.024	0.765 ± 0.102	-0.011
TTA	0.770 ± 0.026	1.000 ± 0.500	0.230

Chill recovery time (min)

cpo haplotype	Prediction ± SE	Observation ± SE	Deviation
CGT	36.88 ± 1.89	41.81 ± 3.45	4.93
CTA	38.14 ± 1.48	35.73 ± 5.04	-2.41
CTT	36.05 ± 1.90	33.93 ± 3.54	-2.12
TGA	36.79 ± 1.90	36.65 ± 4.01	-0.14
TGT	34.70 ± 1.48	28.31 ± 3.31	-6.39
TTT	33.88 ± 0.65	30.25 ± 5.42	-3.62

bioRxiv preprint doi: https://doi.org/10.64898/2026.10.09.758124; this version posted October 11, 2026. The copyright holder for this preprint (which was not certified by peer review) is the author/funder, who has granted bioRxiv a license to display the preprint in perpetuity. It is made available under a CC-BY 4.0 International license.

TTA	35.97 ± 1.89	44.68 ± 5.99	8.71

Lipid mass content (ng)

cpo haplotype	Prediction ± SE	Observation ± SE	Deviation
CGT	58.40 ± 0.96	55.79 ± 4.26	-2.61
CTA	61.02 ± 1.80	60.67 ± 4.45	-0.35
CTT	60.79 ± 1.82	60.54 ± 4.06	-0.25
TGA	60.40 ± 1.82	57.20 ± 4.26	-3.20
TGT	60.17 ± 1.80	60.17 ± 3.93	-0.00
TTT	62.56 ± 1.19	64.49 ± 4.16	1.93
TTA	62.79 ± 0.96	70.78 ± 4.11	7.99

Starvation survival time (hours)

cpo haplotype	Prediction ± SE	Observation ± SE	Deviation
CGT	84.68 ± 2.34	86.86 ± 1.44	2.17
CTA	87.85 ± 1.81	82.10 ± 1.44	-5.75
CTT	88.35 ± 1.41	100.51 ± 1.47	12.16
TGA	82.79 ± 1.41	70.83 ± 1.49	-11.96
TGT	83.29 ± 1.81	81.52 ± 1.46	-1.77
TTT	86.95 ± 2.21	76.57 ± 1.44	-10.39
TTA	86.45 ± 2.34	105.35 ± 1.43	18.89

Heat knockdown time (min)

bioRxiv preprint doi: https://doi.org/10.64898/2026.10.09.758124; this version posted October 11, 2026. The copyright holder for this preprint (which was not certified by peer review) is the author/funder, who has granted bioRxiv a license to display the preprint in perpetuity. It is made available under a CC-BY 4.0 International license.

cpo haplotype	Prediction ± SE	Observation ± SE	Deviation
CGT	74.45 ± 0.92	70.97 ± 3.87	-3.48
CTA	75.13 ± 1.14	73.61 ± 3.70	-1.52
CTT	73.89 ± 0.37	73.22 ± 3.91	-0.67
TGA	77.02 ± 0.37	84.40 ± 3.76	7.38
TGT	75.78 ± 1.14	73.10 ± 4.06	-2.68
TTT	75.23 ± 1.15	77.51 ± 4.44	2.29
TTA	76.46 ± 0.92	74.65 ± 4.02	-1.81

Lifespan (days)

cpo haplotype	Prediction ± SE	Observation ± SE	Deviation
CGT	32.55 ± 1.05	34.26 ± 1.02	1.71
CTA	31.84 ± 1.26	33.80 ± 1.00	1.97
CTT	33.25 ± 0.34	35.76 ± 1.00	2.51
TGA	29.77 ± 0.34	25.87 ± 1.06	-3.90
TGT	31.18 ± 1.26	31.23 ± 1.00	0.05
TTT	31.88 ± 1.25	33.21 ± 1.00	1.33
TTA	30.47 ± 1.05	27.46 ± 1.00	-3.01

Development time (hours)

cpo haplotype	Prediction ± SE	Observation ± SE	Deviation
CGT	223.67 ± 0.72	223.97 ± 1.31	0.31

bioRxiv preprint doi: https://doi.org/10.64898/2026.10.09.758124; this version posted October 11, 2026. The copyright holder for this preprint (which was not certified by peer review) is the author/funder, who has granted bioRxiv a license to display the preprint in perpetuity. It is made available under a CC-BY 4.0 International license.

CTA	222.84 ± 0.82	223.58 ± 1.31	0.73
CTT	224.09 ± 0.42	225.91 ± 1.30	1.83
TGA	222.03 ± 0.42	216.74 ± 1.31	-5.29
TGT	223.28 ± 0.82	227.86 ± 1.30	4.58
TTT	223.70 ± 0.71	221.92 ± 1.32	-1.78
TTA	222.45 ± 0.72	223.34 ± 1.31	0.88

Fecundity (number of eggs)

cpo haplotype	Prediction ± SE	Observation ± SE	Deviation
CGT	38.38 ± 0.19	53.26 ± 5.02	14.88
CTA	34.86 ± 1.95	25.69 ± 5.61	-9.17
CTT	36.66 ± 1.85	35.52 ± 5.29	-1.14
TGA	34.45 ± 1.85	24.44 ± 6.48	-10.01
TGT	36.25 ± 1.95	32.59 ± 5.29	-3.66
TTT	34.53 ± 1.87	30.60 ± 5.29	-3.93
TTA	32.73 ± 0.19	37.99 ± 5.02	5.26

Relative *cpo* expression ($2^{-\Delta Cq}$)

cpo haplotype	Prediction ± SE	Observation ± SE	Deviation
CGT	0.0578 ± 0.0013	0.0589 ± 0.0157	0.0010
CTA	0.0525 ± 0.0039	0.0622 ± 0.0128	0.0097
CTT	0.0552 ± 0.0035	0.0569 ± 0.0128	0.0017

bioRxiv preprint doi: https://doi.org/10.64898/2026.10.09.758124; this version posted October 11, 2026. The copyright holder for this preprint (which was not certified by peer review) is the author/funder, who has granted bioRxiv a license to display the preprint in perpetuity. It is made available under a CC-BY 4.0 International license.

TGA	0.0500 ± 0.0035	0.0561 ± 0.0128	0.0062
TGT	0.0527 ± 0.0039	0.0478 ± 0.0128	-0.0049
TTT	0.0500 ± 0.0035	0.0598 ± 0.0128	0.0098
TTA	0.0473 ± 0.0013	0.0155 ± 0.0128	-0.0318

bioRxiv preprint doi: https://doi.org/10.64898/2026.10.09.758124; this version posted October 11, 2026. The copyright holder for this preprint (which was not certified by peer review) is the author/funder, who has granted bioRxiv a license to display the preprint in perpetuity. It is made available under a CC-BY 4.0 International license.

Table S5. Likelihood-ratio test for the combined effect of three-locus genotype (Combination, 7 levels corresponding to the established ROPs) on each trait, comparing an intercept-only model to a model with Combination as a single categorical predictor. Reported as deviance for the binomial dormancy model, Chisq for traits with a bottle/vial random effect (starvation, lifespan, development), and a Sum-of-Squares-based F-test for all other continuous traits; see Methods. * p<0.05, ** p<0.01, *** p<0.001.

**Trait**	**df**	**Test statistic**	**p**	
Proportion dormant	6	47.85	1.26e-8	***
Chill recovery time (min)	6	1889.63	0.0949	
Lipid mass content (ng)	6	7422.04	0.3612	
Starvation survival time (hours)	6	114.49	2.34e-22	***
Heat knockdown time (min)	6	7028.72	0.2154	
Lifespan (days)	6	55.63	3.46e-10	***
Development time (hours)	6	37.34	1.51e-6	***
Fecundity (number of eggs)	6	5008.59	0.0029	**
Relative cpo expression (2^-ΔCq)	6	4.722e-3	0.1437	

bioRxiv preprint doi: https://doi.org/10.64898/2026.10.09.758124; this version posted October 11, 2026. The copyright holder for this preprint (which was not certified by peer review) is the author/funder, who has granted bioRxiv a license to display the preprint in perpetuity. It is made available under a CC-BY 4.0 International license.

Table S6 Likelihood-ratio test for genotype-combination effects beyond additive main effects, comparing an additive model (Trait ~ cpo^{4408} + cpo^{5558} + cpo^{7866}) to the full three-locus Combination model, for each trait. This isolates the epistatic component specifically, controlling for additive main effects, and complements the vertex-specific deviation tests reported in Table S7. Because the cpo^{CGA} combination is absent from the data, individual 2-way and 3-way interaction terms cannot be separated from one another and this test reports their combined contribution only. * p<0.05, ** p<0.01, *** p<0.001.

Trait	df	Test statistic	p	
Proportion dormant	3	10.12	0.0176	*
Chill recovery time (min)	3	932.97	0.1491	
Lipid mass content (ng)	3	620.45	0.9077	
Starvation survival time (hours)	3	94.94	1.90e-20	***
Heat knockdown time (min)	3	3191.48	0.2863	
Lifespan (days)	3	4.79	0.1878	
Development time (hours)	3	24.41	2.06e-5	***
Fecundity (number of eggs)	3	3252.74	0.0048	**
Relative cpo expression (2^-ΔCq)	3	3.000e-3	0.1077	

bioRxiv preprint doi: https://doi.org/10.64898/2026.10.09.758124; this version posted October 11, 2026. The copyright holder for this preprint (which was not certified by peer review) is the author/funder, who has granted bioRxiv a license to display the preprint in perpetuity. It is made available under a CC-BY 4.0 International license.

Table S7. Deviation of observed trait values from additive null predictions for the tropical (*cpo*^{CGT}) and temperate (*cpo*^{TTA}) three-locus genotype combinations. For starvation resistance, lifespan, and development time, individual values were first averaged to bottle/vial-level means before testing (see Methods); for other continuous traits, individual values were compared directly. Effect sizes are Cohen's h for the binomial dormancy proportion and Cohen's d for all other traits. * p<0.05, ** p<0.01, *** p<0.001.

Proportion dormant

Genotype	Effect size (h)	p		Mean observed	Mean predicted
cpo^{CGT}	-0.143	0.4650		0.605	0.674
cpo^{TTA}	0.999	0.0178	*	1.000	0.770

Chill recovery time (min)

Genotype	Effect size (d)	p		Mean observed	Mean predicted
cpo^{CGT}	0.446	0.2419		41.81	36.88
cpo^{TTA}	1.014	0.1843		44.68	35.97

Lipid mass content (ng)

Genotype	Effect size (d)	p		Mean observed	Mean predicted
cpo^{CGT}	-0.131	0.5607		55.79	58.40
cpo^{TTA}	0.377	0.0880		70.78	62.79

Starvation survival time (hours)

Genotype	Effect size (d)	p		Mean observed	Mean predicted
cpo^{CGT}	0.026	0.9549		86.86	84.68

bioRxiv preprint doi: https://doi.org/10.64898/2026.10.09.758124; this version posted October 11, 2026. The copyright holder for this preprint (which was not certified by peer review) is the author/funder, who has granted bioRxiv a license to display the preprint in perpetuity. It is made available under a CC-BY 4.0 International license.

cpo^{TTA}	5.398	7.33e-7	***	105.35	86.45

Heat knockdown time (min)

Genotype	Effect size (d)	p		Mean observed	Mean predicted
cpo^{CGT}	-0.186	0.3394		70.97	74.45
cpo^{TTA}	-0.046	0.8191		74.65	76.46

Lifespan (days)

Genotype	Effect size (d)	p		Mean observed	Mean predicted
cpo^{CGT}	0.640	0.1864		34.26	32.55
cpo^{TTA}	-1.331	0.0155	*	27.46	30.47

Development time (hours)

Genotype	Effect size (d)	p		Mean observed	Mean predicted
cpo^{CGT}	0.044	0.9240		223.97	223.67
cpo^{TTA}	0.467	0.3240		223.34	222.45

Fecundity (number of eggs)

Genotype	Effect size (d)	p		Mean observed	Mean predicted
cpo^{CGT}	1.449	0.0102	*	53.26	38.38
cpo^{TTA}	0.604	0.2098		37.99	32.73

Relative *cpo* expression ($2^{-\Delta Cq}$)

Genotype	Effect size (d)	p		Mean observed	Mean predicted
cpo^{CGT}	0.036	0.9770		0.0589	0.0578

bioRxiv preprint doi: https://doi.org/10.64898/2026.10.09.758124; this version posted October 11, 2026. The copyright holder for this preprint (which was not certified by peer review) is the author/funder, who has granted bioRxiv a license to display the preprint in perpetuity. It is made available under a CC-BY 4.0 International license.

cpo^{TTA}	-2.490	0.0928	0.0155	0.0473

bioRxiv preprint doi: https://doi.org/10.64898/2026.10.09.758124; this version posted October 11, 2026. The copyright holder for this preprint (which was not certified by peer review) is the author/funder, who has granted bioRxiv a license to display the preprint in perpetuity. It is made available under a CC-BY 4.0 International license.

Table S8. Linkage disequilibrium (D') among the three focal *couch potato* (*cpo*) loci in each wild-collected population, calculated directly from observed 3-locus haplotype counts (not the DGRP genotype-dosage approximation used in Methods/Figure 1). Note that D' at small sample sizes (e.g. Florida, n=13) can be highly sensitive to individual haplotype counts and should be interpreted with caution.

**Population**	**Latitude**	***cpo***^{4408} – ***cpo***^{5558}	***cpo***^{5558} – ***cpo***^{7866}	***cpo***^{4408} – ***cpo***^{7866}
Maine	44.01°N	0.181	0.439	0.350
Pennsylvania	39.12°N	0.004	0.083	0.519
North Carolina	35.5°N	0.136	0.035	0.548
Florida	25.5°N	0.536	1.000	1.000

bioRxiv preprint doi: https://doi.org/10.64898/2026.10.09.758124; this version posted October 11, 2026. The copyright holder for this preprint (which was not certified by peer review) is the author/funder, who has granted bioRxiv a license to display the preprint in perpetuity. It is made available under a CC-BY 4.0 International license.

Table S9. Observed allele and three-locus genotype frequencies in wild-collected populations along the latitudinal cline, compared to predicted frequencies under random assortment of the three focal loci. Predicted genotype frequency is the product of the three single-locus allele frequencies observed in that population. Deviation is observed minus predicted.

Maine (N=79)

	n	Frequency ± SE	Predicted freq.	Deviation
cpo^{4408} C	27	0.342 ± 0.053	—	—
cpo^{5558} G	29	0.367 ± 0.054	—	—
cpo^{7866} T	34	0.430 ± 0.056	—	—
cpo^{CGT}	5	0.063 ± 0.027	0.054	0.009
cpo^{CGA}	8	0.101 ± 0.034	0.071	0.030
cpo^{CTA}	2	0.025 ± 0.018	0.123	-0.098
cpo^{CTT}	12	0.152 ± 0.040	0.093	0.059
cpo^{TGA}	14	0.177 ± 0.043	0.138	0.040
cpo^{TGT}	2	0.025 ± 0.018	0.104	-0.079
cpo^{TTT}	15	0.190 ± 0.044	0.179	0.011
cpo^{TTA}	21	0.266 ± 0.050	0.237	0.029

Pennsylvania (N=110)

	n	Frequency ± SE	Predicted freq.	Deviation
cpo^{4408} C	52	0.473 ± 0.048	—	—

bioRxiv preprint doi: https://doi.org/10.64898/2026.10.09.758124; this version posted October 11, 2026. The copyright holder for this preprint (which was not certified by peer review) is the author/funder, who has granted bioRxiv a license to display the preprint in perpetuity. It is made available under a CC-BY 4.0 International license.

cpo^{5558} G	40	0.364 ± 0.046	—	—
cpo^{7866} T	66	0.600 ± 0.047	—	—
cpo^{CGT}	16	0.145 ± 0.034	0.103	0.042
cpo^{CGA}	3	0.027 ± 0.016	0.069	-0.041
cpo^{CTA}	7	0.064 ± 0.023	0.120	-0.057
cpo^{CTT}	26	0.236 ± 0.041	0.180	0.056
cpo^{TGA}	15	0.136 ± 0.033	0.077	0.060
cpo^{TGT}	6	0.055 ± 0.022	0.115	-0.060
cpo^{TTT}	18	0.164 ± 0.035	0.201	-0.038
cpo^{TTA}	19	0.173 ± 0.036	0.134	0.039

North Carolina (DGRP) (N=145)

	n	Frequency ± SE	Predicted freq.	Deviation
cpo^{4408} C	72	0.497 ± 0.042	—	—
cpo^{5558} G	46	0.317 ± 0.039	—	—
cpo^{7866} T	96	0.662 ± 0.039	—	—
cpo^{CGT}	24	0.166 ± 0.031	0.104	0.061
cpo^{CGA}	2	0.014 ± 0.010	0.053	-0.039
cpo^{CTA}	9	0.062 ± 0.020	0.115	-0.052
cpo^{CTT}	37	0.255 ± 0.036	0.224	0.031
cpo^{TGA}	13	0.090 ± 0.024	0.054	0.036

bioRxiv preprint doi: https://doi.org/10.64898/2026.10.09.758124; this version posted October 11, 2026. The copyright holder for this preprint (which was not certified by peer review) is the author/funder, who has granted bioRxiv a license to display the preprint in perpetuity. It is made available under a CC-BY 4.0 International license.

cpo^{TGT}	7	0.048 ± 0.018	0.106	-0.057
cpo^{TTT}	28	0.193 ± 0.033	0.228	-0.034
cpo^{TTA}	25	0.172 ± 0.031	0.116	0.056

Florida (Homestead) (N=13)

	n	Frequency ± SE	Predicted freq.	Deviation
cpo^{4408} C	4	0.308 ± 0.128	—	—
cpo^{5558} G	6	0.462 ± 0.138	—	—
cpo^{7866} T	10	0.769 ± 0.117	—	—
cpo^{CGT}	3	0.231 ± 0.117	0.109	0.122
cpo^{CGA}	0	0.000 ± 0.000	0.033	-0.033
cpo^{CTA}	0	0.000 ± 0.000	0.038	-0.038
cpo^{CTT}	1	0.077 ± 0.074	0.127	-0.051
cpo^{TGA}	0	0.000 ± 0.000	0.074	-0.074
cpo^{TGT}	3	0.231 ± 0.117	0.246	-0.015
cpo^{TTT}	3	0.231 ± 0.117	0.287	-0.056
cpo^{TTA}	3	0.231 ± 0.117	0.086	0.145

1186

bioRxiv preprint doi: https://doi.org/10.64898/2026.10.09.758124; this version posted October 11, 2026. The copyright holder for this preprint (which was not certified by peer review) is the author/funder, who has granted bioRxiv a license to display the preprint in perpetuity. It is made available under a CC-BY 4.0 International license.

Table S10. Observed allele and three-locus genotype frequencies across seasonal collections (May, June, October) in a Pennsylvania orchard, compared to predicted frequencies under random assortment of the three focal loci within each collection. Predicted genotype frequency is the product of the three single-locus allele frequencies observed in that collection. Deviation is observed minus predicted.

May (N=36)

	n	Frequency ± SE	Predicted freq.	Deviation
cpo^{4408} C	19	0.528 ± 0.083	—	—
cpo^{5558} G	10	0.278 ± 0.075	—	—
cpo^{7866} T	13	0.361 ± 0.080	—	—
cpo^{CGT}	6	0.167 ± 0.062	0.094	0.073
cpo^{CGA}	1	0.028 ± 0.027	0.053	-0.025
cpo^{CTA}	3	0.083 ± 0.046	0.138	-0.054
cpo^{CTT}	9	0.250 ± 0.072	0.244	0.006
cpo^{TGA}	1	0.028 ± 0.027	0.047	-0.020
cpo^{TGT}	2	0.056 ± 0.038	0.084	-0.028
cpo^{TTT}	6	0.167 ± 0.062	0.218	-0.051
cpo^{TTA}	8	0.222 ± 0.069	0.123	0.099

June (N=42)

	n	Frequency ± SE	Predicted freq.	Deviation

bioRxiv preprint doi: https://doi.org/10.64898/2026.10.09.758124; this version posted October 11, 2026. The copyright holder for this preprint (which was not certified by peer review) is the author/funder, who has granted bioRxiv a license to display the preprint in perpetuity. It is made available under a CC-BY 4.0 International license.

cpo^{4408} C	21	0.500 ± 0.077	—	—
cpo^{5558} G	14	0.333 ± 0.073	—	—
cpo^{7866} T	21	0.500 ± 0.077	—	—
cpo^{CGT}	4	0.095 ± 0.045	0.083	0.012
cpo^{CGA}	2	0.048 ± 0.033	0.083	-0.036
cpo^{CTA}	5	0.119 ± 0.050	0.167	-0.048
cpo^{CTT}	10	0.238 ± 0.066	0.167	0.071
cpo^{TGA}	7	0.167 ± 0.058	0.083	0.083
cpo^{TGT}	1	0.024 ± 0.024	0.083	-0.060
cpo^{TTT}	6	0.143 ± 0.054	0.167	-0.024
cpo^{TTA}	7	0.167 ± 0.058	0.167	0.000

October (N=34)

	n	Frequency ± SE	Predicted freq.	Deviation
cpo^{4408} C	17	0.500 ± 0.086	—	—
cpo^{5558} G	13	0.382 ± 0.083	—	—
cpo^{7866} T	14	0.412 ± 0.084	—	—
cpo^{CGT}	4	0.118 ± 0.055	0.112	0.005
cpo^{CGA}	1	0.029 ± 0.029	0.079	-0.049
cpo^{CTA}	3	0.088 ± 0.049	0.127	-0.039
cpo^{CTT}	9	0.265 ± 0.076	0.182	0.083

bioRxiv preprint doi: https://doi.org/10.64898/2026.10.09.758124; this version posted October 11, 2026. The copyright holder for this preprint (which was not certified by peer review) is the author/funder, who has granted bioRxiv a license to display the preprint in perpetuity. It is made available under a CC-BY 4.0 International license.

cpo^{TGA}	7	0.206 ± 0.069	0.079	0.127
cpo^{TGT}	1	0.029 ± 0.029	0.112	-0.083
cpo^{TTT}	6	0.176 ± 0.065	0.182	-0.005
cpo^{TTA}	3	0.088 ± 0.049	0.127	-0.039
